# Heritability of durable resistance to stripe rust in bread wheat (*Triticum aestivum* L.)

**DOI:** 10.1515/biol-2025-1072

**Published:** 2025-07-15

**Authors:** Latifa AlHusnain, Atef Shahin, Farid Mehiar, Kotb A. Attia, Mohamed Eid, Yaser Hafez, Nadi A. Al-Harbi, Khaled Abdelaal

**Affiliations:** Department of Biology, College of Science, Princess Nourah bint Abdulrahman University, P.O. Box 84428, 11671, Riyadh, Saudi Arabia; Wheat Disease Research Department, Plant Pathology Research Institute (PPRI), Agriculture Research Centre (ARC), Kafr Elsheikh, Egypt; Agricultural Botany Department, Faculty of Agriculture, Kafrelsheikh University, 33516, Kafr Elsheikh, Egypt; Center of Excellence in Biotechnology Research, King Saud University, P.O. Box 2455, 11451, Riyadh, Saudi Arabia; EPCRS Excellence Center, Plant Pathology and Biotechnology Laboratory, Agricultural Botany Department, Faculty of Agriculture, Kafrelsheikh University, 33516, Kafr Elsheikh, Egypt; Biology Department, University College of Tayma, University of Tabuk, Tabuk, 47512, Saudi Arabia

**Keywords:** wheat, stripe rust, genetic resistance, inheritance studies, durability

## Abstract

Wheat yellow (stripe) rust is one of the most destructive diseases worldwide. Growing resistant cultivars is the most economical and eco-friendly method to control the disease. To determine heritability and gene number of durable resistance to stripe rust, four Egyptian wheat cultivars were crossed to four genotypes; Jupateco 73R, Opata 85, Anza, and Pavon 76 carrying the durable resistant genes were studied to enhance wheat yellow rust durable resistance. The genetic analysis has been done based on F_1_, and F_2_ of crosses, and our findings confirmed the di-genic and tri-genic control to this disease. The result from the F_2_ population showed that Misr2/Opata and Giza168/Anza were segregated fitting the expected ratios of 57 resistant: 7 susceptible. The segregation ratios of resistance and susceptibility of F_2_ plants showed tri-genic hereditary of yellow rust resistance, permitting the following conclusions about the genes expressed in adult plants suggesting a difference of three dominant genes between the parental cultivars. The cross Giza168/Pavon76 was showed phenotypic ratios fitted the theoretical ratios, 63 resistant: 1 susceptible, suggesting difference of two dominant and one recessive gene for resistance. These genotypes are very important for wheat breeding to stripe rust resistance in Egypt.

## Graphical abstract

**Figure:**
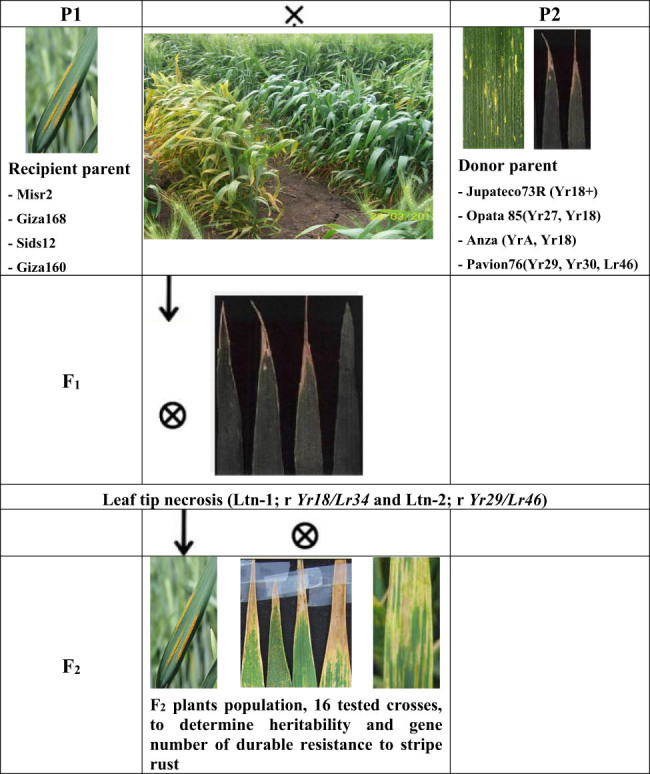

## 1 Introduction

Wheat (*Triticum aestivum* L.) is an essential crop that delivers food to more than 30% of people on earth [1]. Wheat yield and production are influenced by several stress factors [2,3,4,5,6,7]. Among the biotic factors, wheat rusts, i.e., stem rust, leaf rust, and stripe rust, are dramatically harmful grain yield all over the world [8]. Yellow rust caused by *Puccinia striiformis* Westend f. sp. *tritici* [Pst] is one of the most harmful diseases of wheat [9,10,11,12] causing losses in yield [13,14,15,16,17,18]. The appearance and quick spread of new races of yellow rust, i.e., virulence-*Yr9* in 1990, virulence-*Yr27* in 2011, virulence-*Yr32* race in 2014, were recorded in many countries. Recently, the warrior race of wheat Pst with extensive distribution in 2015 has shown a new pathotype of the stripe rust in Egypt. Therefore, some cultivars were recognized to be resistant and become susceptible [19,20]. *Puccinia striiformis* can attack many wheat cultivars, causing yield losses ranged from 10 to 70% and up to 100% in extreme conditions, the highest damage was recorded with wheat genotype; Gemmeiza11, followed by Misr1 then Misr2 and Sids12 [21].

Seventy-five genes with official or temporary symbols have been reported for stripe rust resistance [17,22]. Eighty‐five resistance genes, designated *Yr*, have been known, these genes are dominant, race-specific and so do not deliver durable resistance alone. The durable resistance is a kind of resistance that has continued effective during wheat cultivation for many generations [23]. *Yr18*, *Yr29*, and *Yr46* are the most known genes for durable adult plant stripe rust resistance in hexaploid wheats. *Yr18* and *Yr46* are placed in chromosomes 7D and 4D, respectively, and *Yr29* is located in chromosome 1Bl. A previous study for partial resistance on wheat genotypes was conducted to study *Yr18/Lr34* partial resistance gene to yellow rust expressed in twenty commercial cultivars [24]. Studying the inheritance of rust resistance is very significant in improving rust resistance [25,26,27,28]. Presentation of such a concept in breeding for stripe and leaf rust resistance, commonly known as slow rusting, or partial resistance, has controlled CIMMYT’s wheat genotypes’ improvement for more than 45 years. Our results indicate that durable resistance, early defined by Khan et al. [23], to leaf and stripe rusts of several cultivars is based on the slow rusting genes having additive effects, as a type of genetic resistance that has remained effective in a wheat cultivar, during its widespread cultivation for a long sequence of generations or a long period of time (many years), in a wide range of environments, favorable to disease. Hence, the present work was executed to study the genetic analysis of durable adult plant stripe rust resistance and determine the genes leading to the heredity of resistance to strip rust in four cultivars of bread wheat.

## 2 Materials and methods

### 2.1 Host plants and field experiment

The present work was carried out through the three seasons; 2021, 2022, and 2023 at the experimental farm, Sakha, Kafrelsheikh. Misr2, Sids12, Giza160, and Giza168 cultivars showed different levels of response to yellow rust disease; they were crossed with the wheat genotypes carrying durable resistance genes (resistance parents) to stripe rust disease, i.e., Jupateco73R (*Yr18*), Opata85 (*Yr27*, *Yr18*), Anza (*YrA*, *Yr18*), and Pavon76 (*Yr29*, *Yr30*) to generate F_1_ and F_2_. Egyptian wheat genotypes were crossed and an F_2_ population was generated to study the genetic relationship of adult plant yellow rust resistance possessed by them. Four wheat cultivars were received from the Wheat Department, Agricultural Research Center. The four *Yr* isogenic lines were provided by the International Center ICARDA (Table 1).

**Table 1:** Disease severity of the wheat parents across the years of testing

Genotype	Additive genes for resistance	Field responses across the years of testing			Seed origin
		2021	2022	2023	
Jupateco73R	*Yr18+*	MS	MS	MSS	ICARDA
Opata85	*Yr27*, *Yr18*	MR	MR	MR	ICARDA
Anza	*YrA*, *Yr18*	MS	MRMS	MR	ICARDA
Pavon76	*Yr29*, *Yr30*, *Lr46*	MR	MSS	S	ICARDA
Sids12	Unknown	S	S	S	Egypt
Misr2	Unknown	S	S	S	Egypt
Giza168	Unknown	S	S	MRMS	Egypt
Giza160	Unknown	S	S	S	Egypt

MR, moderately resistant; MRMS, moderately resistant to moderately susceptible; MS, moderately susceptible; MSS, moderately susceptible to susceptible; S, susceptible.

In the first season (2021), the sowing of genotypes was done on three dates to harmonize the difference in the flowering stage. The parent was sown in 2 rows; 3 m long, 16 crosses were planned to yield the F_1_’s hybrid grains. The F_1_ grains were sown in rows 3 m long and 30 cm apart to produce the maximum amount of F_2_ grains in the second season. In the 2023 season, 16 F_1_s, 16 F_2_s, and their 8 parents were set in randomized complete block design for evaluation. The F_1_ and F_2_ were sown in rows 6 m long and 30 cm apart. The plot contains nine rows (one for each for P_1_, P_2_, and F_1_ and six for F_2_). The highly susceptible spreader cultivar (Morocco) was sown as a distributor to spread the urediniospores (Pst).

### 2.2 Field reaction to yellow rust inoculation

The inoculation was done at the booting stage [29]. Urediniospores is virulent for *Yr2*, *Yr6*, *Yr7*, *Yr8*, *Yr9*, *Yr11*, *Yr12*, *Yr17*, *Yr18*, *Yr27*, *YrA+*, and *YrCV* and avirulent for *Yr1*, *Yr5*, *Yr10*, *Yr15*, and *YrSP* genes. The reaction of yellow rust was observed during the adult period when the severity was 30% in the susceptible cultivars of the spreader. The severity (%) was observed from the first time of appearance and every 7 days till the early dough period. The infection type was expressed in five types [30]. The resistance and susceptibility types were near immune (I), resistance (R), moderately resistance (MR), moderately susceptible (MS), and susceptible types (S). R and MR were considered resistance, while MS and S were susceptible. The average coefficient of infection was 0.0, 0.2, 0.4, 0.8. and 1 for I, R, MR, MS, and S types [31]. The ten physiological races listed were identified during the 2022/23 growing seasons, the races used to evaluate the wheat genotypes. Most of these races were virulent to some differentials with the exception of 0E0, while the most virulent race was 246E174, with virulence to 13 of the 17 differential cultivars during the seedling period (Table 1) in the greenhouse. A mixture of Pst pathotypes was used in the field to inoculate the plants of F_1_, F_2_, and F_3_, including the parents, *Yr* differential and near-isogenic lines, and susceptible check Avocet S (Table 2).

**Table 2:** Avirulence and virulence of differential cultivars to distinguish races of Pst during the 2023 growing seasons

Differential cultivars	World and European nomenclature system of Pst races^a^									
	0E0	0E44	4E24	4E60	6E24	6E153	6E166	78E159	174191	246E174
Chinese 166	−^b^	−	−	−	+	−	−	−	−	−
Lee	−	−	+	−	+	+	+	+	+	+
Heines Kolben	−	−	+	+	−	+	+	+	+	+
Vilmorin 23	−	−	−	−	−	−	−	+	+	−
Moro	−	−	−	−	−	−	−	−	−	−
Strubes Dickkopf	−	−	−	−	−	−	−	−	+	−
Suwon 92 × Omar	−	−	−	−	−	−	−	+	−	−
Clement	−	−	−	−	−	−	−	−	+	−
*Tirt. Spelt album*	−	−	−	−	−	−	−	−	−	−
Hybrid 46	−	−	−	−	−	+	−	+	+	+
Reichersberg 42	−	−	−	−	−	−	+	+	+	+
Heine’s Peko	−	+	−	+	+	−	+	+	+	+
Nord Desprez	−	+	+	+	+	+	−	+	+	−
Compair	−	−	+	+	−	+	−	+	+	−
Carstens V	−	+	−	+	−	−	+	−	+	+
Spaldings prolific	−	−	−	−	−	−	−	−	−	−
Heines VII	−	−	−	−	+	+	+	+	+	+
Check; “Morocco”	+	+	+	+	+	+	+	+	+	+

^a^World and European group of wheat differential varieties, the nomenclature system of Pst races [23].

^b^− designates susceptibility of the cultivars and + virulence of the race; *v.* designates susceptibility of the cultivars and virulence of the race.

### 2.3 Statistical analysis

The significant difference between expected and observed ratios in F_2_ populations for yellow rust reaction was observed using the Chi-square test (*χ* ^2^). Chi-square test (*χ* ^2^) was used to test the significance of difference between observed and expected ratios in F_2_ populations for yellow rust reaction. The frequency distributions of the F_2_ populations of the studied crosses were done by dividing the field response into 11 classes, i.e., I, R, 10R, 10MR, 20MR, 10MS, 10S, 30S, 40S, 50S, and 60S. Some genetic parameters were estimates, i.e., means of parents, F_1_ and F_2_, environmental variance estimates (VE) = ([VP1 + VP2 + VF1]/3), phenotypic variance VP = VF2, genotypic variance VG = VP − VE, broad sense heritability (*h*2*b*% = [VG/VP] × 100) [32], the expected genetic advance at 5% selection intensity (∆*g*% = [*k* × (VP)0.5 × *h*2*b*]) [33], and wheat entries coefficient of difference GCV% = ([VG/F2 mean] × 100).

## 3 Results

A field experiment was conducted to study the inheritance of resistance to stripe rust in wheat at the adult stage. Genetic analysis was conducted to determine the inheritance of stripe rust resistance of wheat cultivars. The genetic studies included the evaluation of 8 parents and their F_1_ and F_2_ as infected with a mixture of stripe rust (Pst) physiologic races. Stripe rust response segregation among different crosses is discussed separately. Segregation ratio of resistant to susceptible parents, F_1_ and F_2_ populations were used to assess the number of genes segregation for resistance in the cross.

### 3.1 Responses of wheat genotypes and efficiency of the *Yr* genes

The studied genotypes counting differential hosts and near-isogenic lines to Pst pathotypes showed a wide range of rust responses during the 2021 to 2023 seasons (Table 3). The reaction was different between seedling and the adult plant, whereas genotypes carrying *Yr5* and *Yr15* exhibited high resistance to Pst pathotypes. *Yr1*, *Yr17*, *Yr32*, and *YrSp* became ineffective to the new race, 246E175. These genes were resistant to the previously characterized races. The genotypes with *Yr5*, *Yr10*, and *Yr15* had 0-type or R-type reactions and displayed immune or resistance against the pathogen at the three seasons. The genotypes with *Yr2*, *Yr6*, *Yr7*, *Yr9*, *YrSu*, and *YrA* were susceptible. Conversely, *Yr29*, *Yr18*, and Anza (*YrA + Yr18*) genes were shown in moderately susceptible genotypes.

**Table 3:** Wheat genotypes and reaction to Pst pathotypes produced by yellow rust from 2021 to 2023 seasons

Genotype/Yr gene(s)^a^	Reaction to Pst pathotypes^b^				
	Seedling stage		Adult stage		
	Old pst.	New pst.	2021	2022	2023
Avocet S	9	9	80S	30S	80S
Avocet R)*YrA*)	5	8	60S	30S	60S
Yr1/6*Avoc. (*Yr1*)	0;	3	10S	10S	30S
Yr5/6*Avoc. (*Yr5*)	0	2	0	0	0
Yr6/6*Avoc. (*Yr6*)	8	9	80S	60S	70S
Yr7/6*Avoc. (*Yr7*)	5	9	80S	40S	60S
Yr8/6*Avoc. (*Yr8*)	2	9	R	0	0
Yr9/6*Avoc. (*Yr9*)	5	9	80S	60S	80S
Yr10/6*Avoc. (*Yr10*)	0	6	0	0	10MSS
Yr15/6*Avoc. (*Yr15*)	0	0	0	0	0
Yr17/6*Avoc. (*Yr17*)	2	6	10MSS	30MSS	30MSS
Yr18/6*Avoc. (*Yr18*)	2	8	50MSS	50MSS	30MSS
Yr27/6*Avoc. (*Yr27*)	2	8	20MSS	10S	20MS
Yr32/6*Avoc. (*Yr32*)	0	6	5S	10S	20S
YrSp/6*Avoc. (*YrSp*)	0;	8	0	0	5MSS
Chinese 166 (*Yr1*)	0	0	0	0	TrS
Lee (*Yr7*)	0	9	30S	50S	20S
Heines Kolben (*Yr6 + 1*)	9	9	10S	20S	20S
Vilmorin 23 (*Yr3a*, *4a*)	2	0	5MR	20MR	10S
Moro (*Yr10*)	0	8	0	0	0
Strubes Dickopf (*YrSd*)	0	8	5R	5MR	20MS
Suwon 92/Omar (*YrSu*)	0	8	5R	10MS	5S
Clement (*Yr9*, *2 + ?*)	2	9	10MR	20MR	10MS
Hybrid 46 (*Yr4*)	2	9	0	0	5R
Reichersberg 42 (*Yr7 + ?)*	0	9	0	10MS	10MS
Heines Peko (*Yr6 + ?)*	0	9	5R	10MSS	20MSS
Nord Desprez (*YrNd*)	7	8	0	0	10MR
Compare (*Yr8*)	6	5	5R	10MS	10MS
Carstens V (*Yr32*)	0;	8	5R	5MR	TrS
Spalding Prolific (*YrSp*)	0	5	5MSS	5MS	5S
Heines VII (*Yr2 + ?*)	1	8	5R	5R	30MR
Federation4/Kavkaz (*Yr9*)	3	9	10MSS	20MSS	30S
Trident (*Yr17*)	5	9	50MS	30MSS	20S
Anza (*YrA*, *18*)	2	7	10MRMS	10MS	20MS
Kalyansona (*Yr2*)	2	9	10MSS	5MSS	20MSS
*Triticum spelta album* (*Yr5*)	0	0	0	0	0
TP1295 (*Yr25*)	0	9	30S	20S	30S
Jupateco “R” (*Yr18+*)	5	7	5MSS	50MSS	60MSS
Fielder (*Yr6*, *Yr20*)	9	9	30S	40S	40S
Lemhi (*Yr21*)	2	7	30S	50S	60S
LalBahadur/Pavon BL (*Yr29*)	2	9	5MS	20MS	30MS
Opata 85 (*Yr27*, *Yr18*)	2	8	20MS	20MS	30MS
Ciano 79 (*Yr27*)	2	6	30MSS	30MSS	60MS
*Yr28*/Avoc. (*Yr28*)	0	6	30S	30S	60S
Pavon 76 (*Yr29*, *Yr30*)	0	8	30MS	30MS	40MS
Pastor (*Yr31*, *APR*)	0	8	50MSS	20MSS	30MSS
Morocco	9	9	80S	90S	90S

0 = Immune. R = resistant; MR = moderately resistant; MS = moderately susceptible and S = susceptible.

^a^Resistance genes [13].

^b^ITs based on Roelfs et al. [34].

### 3.2 Responses of parents and F_1_ wheat genotypes

The resistance to Pst pathotypes was detected among the parental local varieties and parental carrying stripe rust resistance genes during the adult period in the field across the years of testing. There was intensive disease stress during the three seasons in the field. The four genotypes carrying stripe rust resistance (*Yr*) genes: Jupateco 73R (*Yr18*), Opata85 (*Yr27*, *Yr18*), Anza (*YrA*, *Yr18*), and Pavon76 (*Yr29*, *Yr30*, *Lr46*) showed stripe rust severity and infection responses ranging from trace responses moderately resistance (MR) to moderately susceptible to susceptible (MSS) responses during the 2021, 2022, and 2023 screening experiments at Sakha. The parents’ cultivars: Misr2, Sids12, Giza160, and Giza168 showed a reaction of susceptible (S) during the adult period in the field in Table 1.

**Figure 1:**
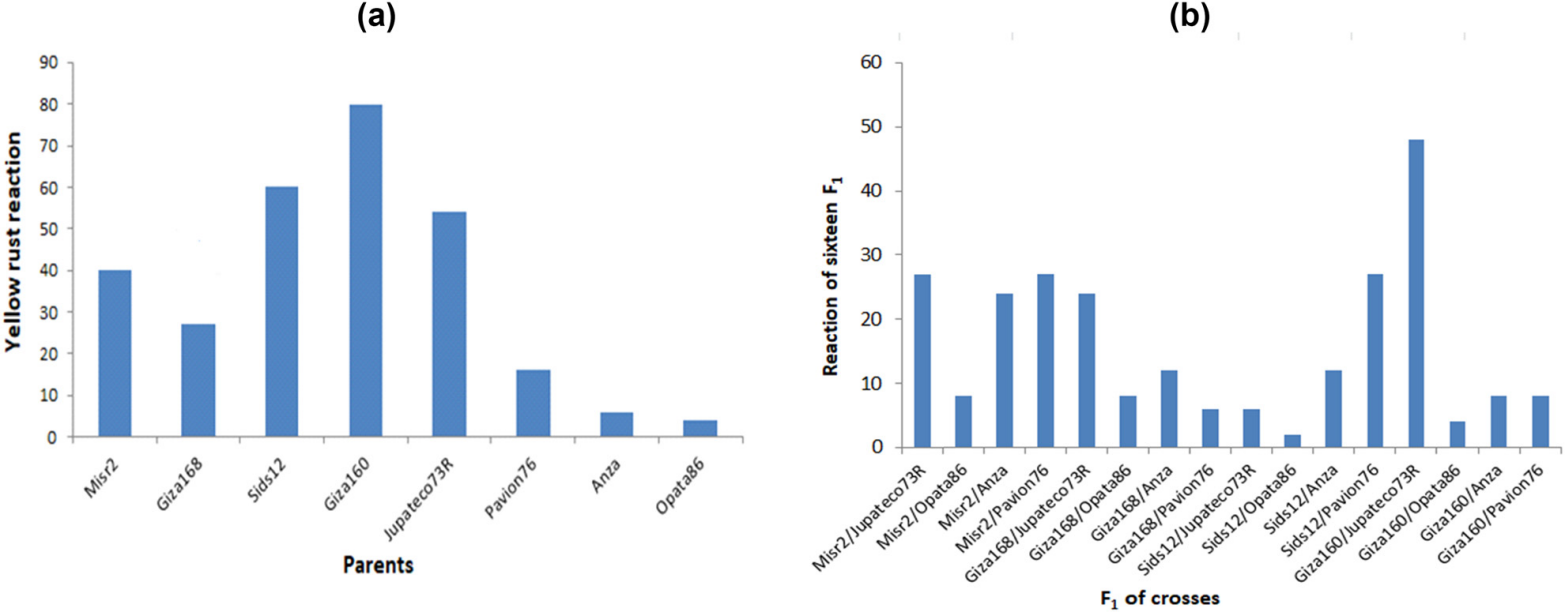
Yellow rust reaction (a) 8 parents, 4 Egyptian bread wheat cultivars and 4 wheat genotypes carrying stripe rust resistance genes, and (b) 16 F_1_ crosses.

Three Egyptian wheat cultivars, i.e., Misr2, Sids12, and Giza160, are susceptible (S) while the wheat cultivar, Giza 168, displayed a response of MSS to Pst populations at the APR. On the other hand, the four isogenic parents, having stripe rust resistance genes, showed 60MSS, 10MR, 10MRMS, and 20MS, disease field response, respectively. The F_1_-tested plant showed moderate resistance to moderately susceptible to susceptible responses. Opata 86 crosses with the four Egyptian wheat varieties displayed MR to yellow rust at APR in adult plants under field conditions except for the cross Sids 12/Opata 86, which was the resistance (R) (Table 4 and Figure 1).

**Table 4:** The reaction to yellow rust under field conditions

Cross name	Adult plant reaction to yellow rust		
	P_1_	P_2_	F_1_
Misr2/Jupateco73R	40S	60MSS	30MSS
Misr2/Opata86	40S	10MR	20MR
Misr2/Anza	40S	10MRMS	30MS
Misr2/Pavion76	40S	20MS	30MSS
Giza168/Jupateco73R	30MSS	60MS	30MS
Giza168/Opata86	30MSS	10MR	20MR
Giza168/Anza	30MSS	10MRMS	20MRMS
Giza168/Pavion76	30MSS	20MS	10MRMS
Sids12/Jupateco73R	60S	60MSS	10MRMS
Sids12/Opata86	60S	10MR	10R
Sids12/Anza	60S	10MRMS	20MRMS
Sids12/Pavion76	60S	20MS	30MSS
Giza160/Jupateco73R	80S	60MSS	60MS
Giza160/Opata86	80S	10MR	10MR
Giza160/Anza	80S	10MRMS	10MS
Giza160/Pavion76	80S	20MS	10MS

### 3.3 The field response of F_2_ populations

F_2_ populations segregated for stripe rust resistance, the Chi-square tests exposed that the segregation results gave a good fit for segregation at three, two, or one independent loci. Data presented in Table 5 showed that the sixteen studied crosses at adult plants under field conditions. Concerning, the second-generation plant populations, eleven of sixteen crosses were resistant.

**Table 5:** Response of sixteen wheat F_2_ populations at adult stage for stripe rust under inoculation with Pst during 2023 season

Cross	No. of plants			Hypothetical ratio	Chi square (*χ* ^2^)	Hypothesized number of genes*
	*R*	*S*	Total			
Misr2/Jupateco 73R	170	40	210	13:3	0.0122	1R, 1D, independent
Misr2/Opata 85	180	25	205	57:7	0.5990	3D
Misr2/Anza	210	10	220	15:1	1.0909	2R, independent
Misr2/Pavion76	170	45	215	13:3	0.6708	1R, 1D, independent
Giza168/Jupateco73R	35	180	215	3:13	0.8617	1R, 1D
Giza168/Opata85	183	18	201	15:1	2.4600	2D, complementary
Giza168/Anza	205	15	220	57:7	0.300	3D
Giza168/Pavion76	190	25	215	61:3	0.0100	2D, 1R
Sids12/Jupateco73R	130	80	210	9:7	2.7286	2D
Sids12/Opata85	150	70	220	11:5	0.0331	1R, 1D
Sids12/Anza	145	65	210	11:5	0.0087	1R, 1D
Sids12/Pavion76	90	125	215	7:9	0.3119	2R
Giza160/Jupateco 73R	95	125	220	7:9	0.0289	2R
Giza160/Opata85	142	68	210	11:5	0.1250	1D, 1R
Giza160/Anza	50	170	220	1:3	0.6061	1R
Giza160/Pavion76	80	125	205	7:9	1.8602	2R

*D = dominant and R = recessive.

The rest of crosses, i.e., Giza168/Jupateco73R, Sids12/Pavion76, Giza160/Jupateco73R, Giza160/Anza, and Giza160/Pavion76, were separated in ratios ranging for susceptible infection type, which fitted the expected ratios of 3R:13S, 7R:9S, 7R:9R, 1R:3S, and 7R:9S; these commercial cultivars when crossing with carrying stripe rust resistance genes (resistance parents) do not have these genes inside Egyptian cultivars. In crosses, Sids12/Pavion76, Giza 160/Jupateco73R, and Giza160/Pavion76 F_2_ segregation ratios were 7R:9S indicating that there are two recessive genes to the tested races. In addition, in cross Giza160/Anza, F_2_ segregation ratios were 1R:3S indicating the genetic control by a recessive gene, while, in cross Giza160/Anza, F_2_ segregation ratios were 3R:13S indicating that there was one recessive gene and one dominant gene. The F_2_ adult plants came from crosses of Misr2 cultivar with each of Jupateco73R, Opata86, and Pavion76 showing resistance to yellow rust and segregated into 170R:40S, 180R:25S, 210R:10S, and 170R:45S plants, with expected ratios 13:3, 57:7, 15:1, and 13:3, respectively. Ratios of resistant and susceptible F_2_ plants showed digenic and trigenic inheritance of stripe rust resistance (Table 3). Crosses of Giza168 with each of four *Yr* monogenic lines displayed resistance to yellow rust except for Giza168/Jupateco73R and segregated into 35R:180S, 183R:18S, 205R:15S, and 190R:25S plants, with expected ratios 1:13, 15:1, 57:7, and 61:3, respectively. The F_2_ segregation ratios for Giza168/Pavion76 show that there are two dominant genes and one recessive gene for resistance against the dominating Pst races in Egypt.

**Figure 2:**
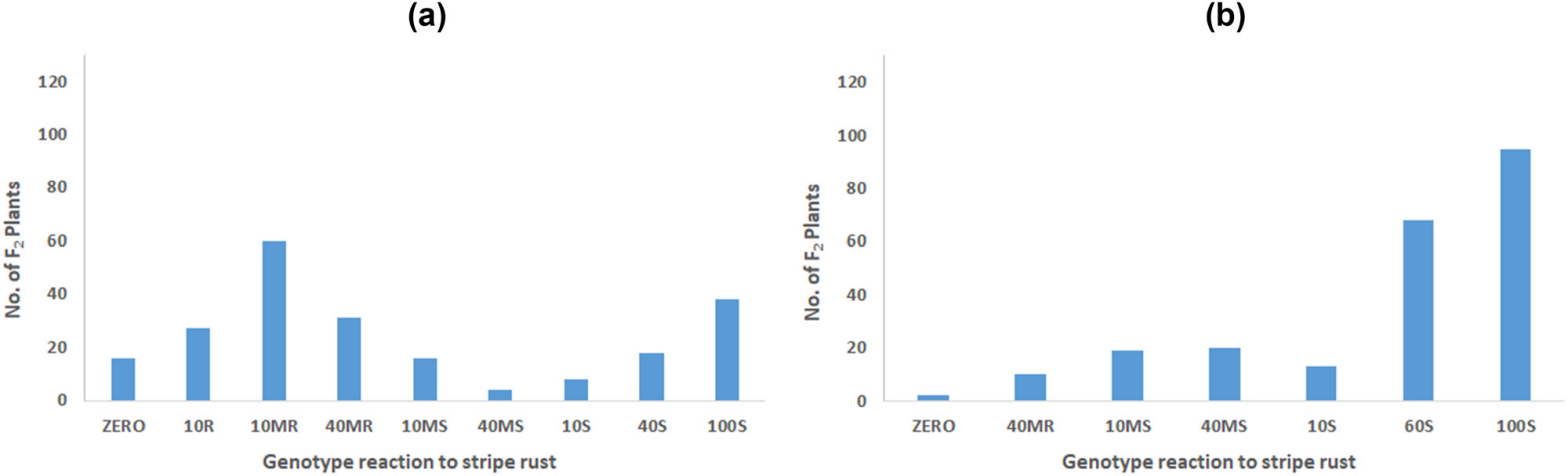
Yellow rust reaction of the two F_2_ crosses for both (a) Misr2 and (b) Giza160 wheat cultivars with the cultivar carrying stripe rust resistance genes; Jupateco 73R (*Yr18*).

In Table 3, crosses of Sids12 with each of four *Yr* monogenic lines displayed resistance to yellow rust except for Giza168/Pavion76. The F_2_ segregation ratios for Giza168/Pavion76 segregated into 90R:125S with expected ratios 7:9 show that there are two recessive genes. On the other hand, the same cross of Sids12 with Jupateco73R separated as 130 resistant and 80 susceptible plants, which gave a fit in 9 resistant (R):7 susceptible (S) ratio, indicating that there are two dominant genes with complementary interaction for resistance to the tested races. The F_2_ population derived from Giza160/Opata85 segregated as 142 resistant and 68 susceptible plants, which gave a good fit in 11 resistant: 5 susceptible ratio (Table 5) thereby suggesting digenic inheritance with a dominant and recessive gene for resistance to the tested pathotypes of stripe rust. The F_2_ adult plants came from a cross of Misr2 cultivar with Jupateco73R segregated into 170R:40S which gave a fit in 9 resistant (R): 7 susceptible (S) ratio, indicating the digenic inheritance with dominant and recessive genes. The F_2_ segregation ratios for Giza160/Jupateco73R segregated into 95R:125S with expected ratios 7:9 show that there are two recessive genes. (Table 5 and Figure 2).

### 3.4 Genetic characterization

Genetic characterization was done based on ACI values. ACI mean values of P_1_ ranged from 23.80 for Giza168 to 79.80 for Giza 160; while, ranged from 3.90 for Opata 85 to 54.00 for Jupateco73R of second parent (P2); from 2.0 for F_1_ of the cross Giza160/Jupateco73R to 47.80; from F_1_ of the cross Misr2/Jupateco73R; from 2.12 for the F_2_ population of the cross Giza168/Anza to 65.24 for F_2_ population of the cross Giza160/Jupateco 73R (Table 6).

**Table 6:** Genetic characters based on ACI for yellow rust of sixteen crosses

Cross	Genetic parameter									
	Mean				Variance			*h* ^2^ *b*%	∆*g*%	GCV%
	P_1_	P_2_	F_1_	F_2_	VP	VE	VG			
Misr2/Jupateco73R	39.60	47.80	54.00	16.62	845.18	0.30	844.89	99.96	59.87	174.90
Misr2/Opata85	39.60	7.70	7.70	8.05	348.68	0.24	348.44	99.93	38.44	231.88
Misr2/Anza	39.60	5.60	23.90	4.45	181.95	0.29	181.66	99.84	27.74	302.88
Misr2/Pavion76	39.60	17.60	27.30	16.41	949.48	0.26	949.23	99.97	63.46	187.81
Giza 168/Jupateco73R	23.80	53.90	23.80	14.24	641.08	0.23	640.85	99.96	52.14	177.84
Giza 168/Opata85	23.80	4.00	8.00	9.26	369.06	0.06	369.00	99.98	39.57	207.44
Giza 168/Anza	23.80	6.00	12.00	2.12	11.82	0.06	11.77	99.50	7.05	161.80
Giza 168/Pavion76	23.80	8.00	6.00	33.02	1555.01	0.06	1554.96	100.00	81.23	119.42
Sids12/Jupateco73R	59.80	54.00	5.90	27.65	1363.01	0.17	1362.85	99.99	76.04	133.54
Sids12/Opata 85	59.80	3.90	3.00	20.81	1091.54	0.09	1091.45	99.99	68.05	158.79
Sids 12/Anza	59.80	6.10	12.00	20.71	1029.59	0.17	1029.43	99.98	66.09	154.92
Sids 12/Pavion76	59.80	17.80	26.80	38.63	1613.51	0.18	1613.34	99.99	82.74	103.99
Giza 160/Jupateco73R	79.80	53.80	2.00	65.24	1388.94	13.45	1375.49	99.03	76.03	56.85
Giza 160/Opata 85	79.80	3.80	3.80	21.01	1024.05	0.18	1023.87	99.98	65.91	152.30
Giza 160/Anza	79.80	5.80	7.70	54.48	1659.04	0.20	1658.85	99.99	83.90	74.77
Giza 160/Pavion76	79.80	17.80	7.70	53.53	1656.61	0.20	1656.42	99.99	83.84	76.03

VP, VE, and VG = phenotypic, environment, and genetic variances, respectively, *h*2*b* = broad sense heritability, ∆*g*% = the expected genetic advance under selection, and GCV% = genotypic coefficient of variation.

Regarding variance estimates (VE), environmental phenotypic (VP), and genotypic (VG) variances reached from 11.82, 0.06, and 11.77 for the cross Giza168/Anza to 1,656.61, 0.20, and 1,656.42 for Giza160/Pavion76, respectively. Broad sense heritability (*h*2*b*%) ranged from 99.03 for Giza160/Jupateco73R to 100.0 for Giza168/Pavion76. The genetic advance from selection (∆*g*%) ranged from 7.05 for Giza186/Anza to 83.90 for Giza160/Anza. However, GCV% ranged from 74.77 for Giza 160/Anza to 302.88 for Misr2/Anza (Table 6).

## 4 Discussion

Breeding approaches of the resistant cultivars to wheat rusts, based on the utilization or the use of durable resistance, are similarly effective against most pathogen races (race-non-specific resistance). It is known to be long-lasting, for many years under wide cultivation in different environmental conditions and hopes to be more durable [28]. The study of durable adult plant stripe rust resistance is very important for enhancing the resistance to yellow rust.

The F_1_ plants’ field response displayed dominance of MR to MS; therefore, they were considered to be durability-resistant cultivars in all crosses to yellow rust except for Sids12/Opata 86, dominant of resistance (Table 2). The durable resistance expressed in the F_1_ tested plant of fifteen crosses, owing to resistance among parents, which were carries of the durable resistance gene such as *Lr34* and *Lr46* and the linked resistance genes *Yr18* and *Yr29*, are linked with durable resistance to the two diseases [35]. The crosses between the susceptible parents Giza160 with the four *Yr* isogenic lines displayed durable resistance in Giza160/Opata85 representing the effectiveness of *Yr27*, *Yr8 + APR* gene conferring resistance to Pst. The results of F_2_ segregation ratios of Giza160/Opata85 cross showed that one dominant and one gene recessive controlling resistance in the crosses Giza160/Opata85, with segregation ratios of 11:5. Regarding the crosses between Giza160 with the same four *Yr*s, the data showed that two complementary recessive genes found to be conferring resistance in Giza160/Jupateco73R and Giza160/Pavion76 with segregation ratios 7:9. One recessive gene pairs led to control the resistance in Giza 160/Anza with 1:3 segregation ratio. APR is regularly based on two or more recessive genes with additive impact which may imply that this type of APR is durable. The F_2_ segregation ratio from two crosses Misr2/Opata85 and Giza168/Anza tested indicates that there are three dominant genes, with segregation ratios 57:7.

Our results also showed that the resistance to this disease is controlled by partial dominance or recessive, and these findings agreed with the result of Singh et al. [36]. They found that the susceptible cultivars have 4 or 5 durable resistance genes, Carstens V/Hybrid 46 confirmed with race CDL-21 display that there are 4 resistance genes in the progeny [37]. The genetic variance represented the majority of the total variance; this result is consistent with earlier studies and demonstrated a high estimate of inheritance and the anticipated advancement of genes under selection in most crosses [27,28,38,39]. Most cultivars have low levels of resistance to yellow rust because of the emergence of new races and virulence changes of the wheat yellow rust disease [20]. The breeding for durable resistance commonly known as slow rusting or partial resistance. Our results indicate that durable resistance, early defined by Khan et al. [23] to stripe rusts of several wheat genotypes is based on the slow rusting genes having additive effects, as a type of genetic resistance that has remained effective in wheat genotypes, during its widespread cultivation for a long sequence of generations or a long period of many years, in a wide range of environments. These lines can be used as a source of durable resistance genes for wheat breeding to stripe rust resistance as well as promoted to the national wheat yield trials for release as new.

## 5 Conclusions

Our results concluded that the application of resistant cultivars is the most economical and eco-friendly technique to manage wheat yellow rust in wheat (*T. aestivum* L.). Four wheat cultivars were crossed to four genotypes: Jupateco 73R, Opata 85, Anza, and Pavon 76 carrying the durable resistant genes to enhance wheat yellow rust durable resistance. The result displayed that Misr2/Opata and Giza168/Anza were segregated fitting the expected ratios of 57 resistant:7 susceptible. Also, the cross Giza168/Pavon76 showed phenotypic ratios fitted the theoretical ratios, 63 resistant: 1 susceptible, suggesting difference of two dominant and one recessive gene for resistance. Generally, these wheat genotypes can be used as a source of durable resistance genes for wheat breeding to stripe rust resistance as well as provide material support and a theoretical basis for control of wheat stripe rust.
